# Mapping Japan’s China-related twitter discourse (2010–2024) using BERTopic

**DOI:** 10.1371/journal.pone.0343085

**Published:** 2026-02-18

**Authors:** Shanshan Zhang, Xi Chen

**Affiliations:** 1 Graduate School, Xi’an International Studies University, Xi’an, China; 2 School of Japanese Culture and Economics, Xi’an International Studies University, Xi’an, China; Universidad Diego Portales, CHILE

## Abstract

Given China’s profound influence on Japan, Japanese public opinion toward China has been the focus of debate. Yet little is known about how such perceptions evolve over time within the digital public sphere. Drawing on a dataset of over one million China-related tweets from Japan (2010–2024), this study integrates BERTopic topic modeling with large language model–driven sentiment analysis to trace the dynamic evolution of Japanese perceptions of China. Empirical analyses find that: (1) Public attention to China has steadily increased, concentrating on four domains: diplomacy & security, environment & health, economy & trade,and culture & society; (2) overall sentiment is predominantly negative, with neutral and positive attitudes appearing in specific topics; and (3) topic–sentiment linkage analysis reveals divergent affective tendencies across topics, indicating that public opinion evolves in response not only to external events but also to the inherent characteristics of topics. By applying computational analysis to large-scale social media data, this study uncovers the dynamic structure of Japanese public opinion regarding China, offering insights into the mechanisms of opinion formation with implications for Sino-Japanese relations. Methodologically, it contributes innovative approaches to the analysis of transnational public discourse.

## Introduction

China and Japan, the world’s second- and third-largest economies, play a pivotal role in shaping regional security and global prosperity. Bilateral relations not only affect their domestic affairs but also exert significant influence on the stability of East Asia and beyond. Yet Japan’s long-standing “U.S.-centred” foreign policy tradition, coupled with the rise of anti-China sentiment since the mid-2000s, has made the relationship increasingly complex [1]. More recently, the COVID-19 pandemic, the intensification of U.S.–China rivalry, and China’s rapid rise have forced Japan to reconsider its geostrategic positioning and pursue regional diplomacy to ease strategic anxieties [2].

Against this backdrop, public perceptions of China have become a crucial factor in shaping Japan’s foreign policy calculus. In representative democracies, public opinion both constrains policy choices and provides critical cues for political decision-making [3–5]. Understanding how the Japanese perceive China is thus central to explaining the social foundations of Sino-Japanese relations and anticipating future policy orientations. Prior studies have examined the effects of state policy, territorial disputes, major international events, and media narratives on Japanese attitudes toward China [6–10]. Yet such research—largely reliant on survey data or short-term event-driven observations—tends to reduce public opinion to a binary “positive–negative” frame [11–13]. As a result, gaps remain in comprehensively analysing the nuances of public perceptions and their temporal evolution.

Social media platforms such as Twitter have become central arenas for public expression and political debate [14,15], providing unique opportunities to observe Japanese views of China [16,17]. Compared to traditional opinion polls, social media data are larger in scale, more dynamic, and more diverse [18]. Fine- grained analyses can thus capture real-time fluctuations in sentiment and reveal the thematic structures underlying public discourse. Despite these advantages, few studies have leveraged social media to systematically trace the long-term dynamics of Japanese public opinion toward China.

To address this gap, this study applies large language model-based topic modeling and sentiment analysis to more than one million China-related tweets posted in Japan between 2010 and 2024. Specifically, it seeks to answer two research questions:

RQ1: What thematic patterns characterize Japanese social media discourse on China, and how have they evolved over time?

RQ2: What sentiment orientations toward China emerge in these discussions, and how do they vary across topics and over time?

By combining methodological innovation with longitudinal scope, this study contributes in three ways. Empirically, it offers a fine-grained mapping of Japanese discourse on China across a fifteen-year period. Methodologically, it demonstrates the utility of large-scale computational approaches for integrating topic structures and sentiment dynamics. Theoretically, it deepens our understanding of how digital public opinion interacts with foreign policy, offering insights into the societal underpinnings of Sino-Japanese relations and, more broadly, the role of public sentiment in international politics. Together, these contributions highlight the value of computational social science approaches in capturing the complex, evolving relationship between public opinion and foreign policy.

## Related work

The literature review is organized around three aspects. First, it systematically surveys existing scholarship on Japanese public opinion toward China. Second, it reviews the methodological advances in topic modeling within social media research. Third, it offers a comprehensive assessment of studies on sentiment analysis in social media, highlighting both their strengths and limitations.

### Research on Japanese public opinion toward China

Japanese perceptions of China have profound implications for bilateral relations and policymaking [19]. Existing research has largely focused on elite-level dynamics, including diplomatic interactions, economic policy, and mainstream media narratives, examining how these actors shape public perceptions and issue framing. For example, Yoshimatsu finds that Japan’s response to the Belt and Road Initiative combines normative frameworks such as “quality infrastructure” with narrative diplomacy, reflecting efforts to maintain international influence amid relative power shifts [2,20]. Media discourse studies further suggest that Japanese coverage of the Belt and Road Initiative exhibits caution and skepticism, which may exacerbate bilateral tensions and negatively influence public attitudes [10,21]. On specific policy issues, Shao emphasizes that discourse about China in Japan reflects competition among multiple political actors rather than a single national narrative [9], while Chen & Zhang highlight a strategic shift in Japan’s China policy from traditional “hedging” toward a dual-track approach balancing economic engagement with security concerns [22].

At the public-perception level, studies have predominantly employed survey methods to examine impressions, attitudes, and policy preferences toward China. Research generally indicates that Japanese attitudes toward China have become increasingly negative [6,11,13,23], shaped by an interplay of factors such as geopolitical tensions, cultural proximity, historical memory, and perceived security threats [6,11,12]. Unlike the economically oriented evaluative patterns observed in many developing countries, the Japanese public tends to view China through an ideological lens [13]. Empirical analyses further suggest that Japanese attitudes toward China are influenced not only by economic expectations but are particularly sensitive to perceived political threats [7]. Moreover, China’s promotion of the “peaceful rise” narrative has been identified as a major factor exacerbating negative perceptions [23].

Recently, a growing body of research has turned to social media data to investigate the dynamics of China-related public opinion during specific events. For instance, in international debates over Japan’s nuclear wastewater discharge, China was framed as the primary opposing actor [24]. During the COVID-19 pandemic, China remained central to Japanese social media discourse, with public sentiment characterized predominantly by anxiety and concern, although emotional intensity moderated after April 2020 [8].While these studies demonstrate the potential of social media for capturing public opinion dynamics, they remain event-specific and cross-sectional, offering limited insight into long-term or evolving patterns of sentiment. As a result, the longitudinal dynamics of Japan-related digital discourse on China remain underexplored.

### Topic modeling in social media research

Social media content presents unique analytical challenges due to its fragmented nature, hierarchical topic structures, and multidimensional expressions [25]. For extracted Twitter data, Latent Dirichlet Allocation (LDA) is a commonly used topic modeling method that has long been applied to identifying public opinion topics and their temporal dynamics [26].However, this approach typically suffers from issues such as semantic ambiguity of topics, limited ability to process multilingual and noisy text, and poor performance with short texts [27], making it difficult to extract valuable information from social media data and prone to overlooking niche yet significant topics [18].

With the rapid advances in machine learning, BERTopic-based topic modeling has emerged as a method capable of extracting dynamically evolving topic units with high semantic precision, automatically generating hierarchical topic structures, and offering flexible combinations of model components [28]. This approach not only accurately captures mainstream discourse topics but also highlights marginalized or overlooked voices, making it particularly suitable for handling unstructured, short-text, and multilingual social media data [29–33]. For instance, Murayama et al. analysed over 6.8 million tweets across 14 languages worldwide, demonstrating the method’s adaptability for cross-hierarchical and multilingual analysis [29]. Veigel et al. tracked shifts in public cognition in German tweets surrounding flooding events since 2014, highlighting BERTopic’s strengths in modeling dynamic topic evolution [30]. The widespread application of BERTopic to large-scale multilingual datasets and event-specific social media analysis underscores its precision for large-scale language analytics. These advances indicate that BERTopic is a powerful tool for analysing and exploring long-term, dynamic patterns in public discourse.

### Sentiment analysis

Sentiment analysis represents another key dimension in social media research, aiming to uncover the distribution and temporal evolution of public attitudes and emotions. Early lexicon-based methods inferred polarity and intensity from sentiment-laden words [34,35], performing adequately on small annotated corpora but struggling with contextual ambiguity and nuanced semantic interpretation. Subsequently, researchers employed traditional machine learning methods based on feature engineering, such as support vector machines and logistic regression, to improve classification performance, yet these approaches are constrained by heavy reliance on manually constructed features and limited generalizability [36].

In recent years, neural network-based pre-trained language models have demonstrated remarkable efficacy in tweet sentiment classification [37,38]. BERT-based sentiment analysis, in particular, substantially improves accuracy by capturing contextual dependencies and deep semantic representations [39,40]. Empirical evidence consistently shows that BERT and its variants outperform traditional methods in multilingual social media sentiment analysis [41–44]. However, most existing research has focused on English or Spanish, and sentiment studies on Japanese public social media discourse remain relatively scarce.

Moreover, recent research highlights the significant role of demographic characteristics in shaping emotional expression on social media. Gender, in particular, has been consistently shown to affect the intensity of emotional expression, engagement with toxic content, and persuasive outcomes [45,46]. This body of research underscores that online emotional expressions and interaction patterns are shaped not only by textual content and semantic structures but also by user attributes. These findings provide an important contextual foundation for understanding the distribution of emotions and interaction mechanisms in social media environments.

In summary, previous research on Japanese public opinion toward China mainly fell short in three aspects. First, the longitudinal evolution of Japanese attitudes toward China remains insufficiently studied, particularly in digital discourse. Second, social media studies have often been limited to single events or cross-sectional snapshots. Third, methodological tools such as topic modeling and sentiment analysis, while promising, have seen limited application to Japanese data and are rarely integrated to capture dynamic, multi-dimensional patterns of discourse. Building on a large-scale dataset of Japan-related tweets, this study offers an in-depth examination of topic evolution and sentiment polarization, providing novel theoretical perspectives and empirical insights for understanding Japanese public opinion toward China.

## Data and methodology

**Fig 1:**
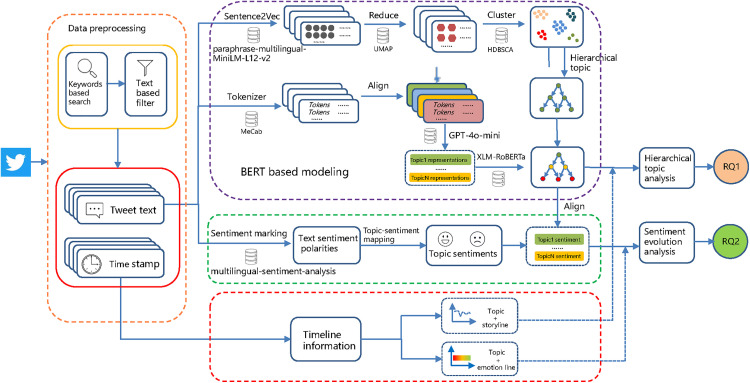
Overview of research framework from twitter data collection to the analysis of topic and sentiment dynamics.

This section introduces the data and research methods, including the establishment of the dataset, construction of hierarchical topic structures, the analysis of topic and sentiment evolution. Fig 1 presents the overall research framework, illustrating the process from Twitter data to topic and sentiment evolution analysis.

### Data construction

Building on the function of hashtags in delineating discursive communities on social media [47], this study uses “#中国” and “#中華” as search keywords to systematically capture the contours of Japanese public discussions concerning China. Prior studies suggest that, in public discourse on foreign-related issues, country names frequently serve as salient linguistic markers that explicitly signal the focal object of discussion and structure discursive meaning [48]. Building on this insight, this study employs highly recognizable country-name labels as the primary entry point for large-scale data collection. This strategy allows for a clear operational delineation of the research object while capturing the overwhelming majority of China-related discussions among Japanese users, and is therefore unlikely to introduce systematic bias into the results. Using the Twitter API, all Japanese-language tweets containing these hashtags between January 1, 2010, and December 31, 2024, were collected, resulting in a 15-year corpus comprising over one million entries (N = 1,310,786). The decision to select 2010 as the starting point is based on the incident in which Japan detained a Chinese fishing vessel near the Diaoyu/Senkaku Islands, a watershed moment that marked the onset of a new phase of strategic competition and frequent tensions in Sino-Japanese relations. This turning point inevitably exerted a profound influence on public discourse practices. Accordingly, the present study adopts 2010 as the baseline, analysing 15 years of public discussions to capture the thematic and emotional dynamics of Japanese discourse on China.

The retrieved tweets included user ID, posting time, user nickname, user name, tweet content, number of likes, and retweet counts. Tweets were classified into four main types: original tweets, retweets, quote tweets, and reply tweets. All original tweets were retained as independent entries in the corpus. Retweets, being semantically identical to the original tweets, can create an “amplification effect” that disproportionately affects topic distributions; to maintain the stability of topic representation, retweets were removed. Quote tweets, by contrast, often include user-added commentary when referencing the original tweet, contributing additional semantic content; such commentary was extracted and treated as a separate entry. Reply tweets, given their interactive and often brief, colloquial nature, were also excluded from the corpus.

It should be noted that this study aims to identify China-related discussion topics among the Japanese public. However, as Twitter has continuously strengthened user privacy protections, accurate location information is unavailable for the vast majority of tweets. Accordingly, following a commonly adopted proxy approach in social media research, we define the analytical object as “China-related tweets written in Japanese.” This approach may introduce a limited number of tweets authored in Japanese by non-Japanese users; however, the proportion of non-native users tweeting in Japanese is relatively small and unlikely to bias the results of this study.

For the raw data, duplicate tweets were merged and removed based on user ID and posting time, and completely identical tweets were deleted, resulting in a final dataset of 1,099,786 valid tweets.

The preprocessing procedure consisted of three steps. First, data cleaning was conducted to remove HTML tags, URLs, emojis, and extraneous whitespace, while normalizing full-width and half-width characters. Second, the tweet text and corresponding timestamps were retained, with key information systematically extracted. Third, Japanese text was segmented using the MeCab tokenizer, which integrates authoritative dictionaries including IPA, UniDic, and Juman. Owing to its extensive dictionary coverage and support for user-defined lexicons, MeCab delivers precise tokenization even in complex linguistic contexts.

### Methodology

To address the two research questions, we conducted hierarchical topic extraction, topic evolution analysis, and sentiment evolution analysis.

#### Hierarchical topic extraction.

This study employed BERTopic for constructing a hierarchical topic model. The detailed procedure is outlined as follows:

(1) Pre-processed tweets were first embedded using the paraphrase-multilingual-MiniLM-L12-v2 model [49], a sentence-transformer that generates embeddings at the sentence level to preserve semantic integrity. In this study, we set the max_length parameter of the paraphrase-multilingual-MiniLM-L12-v2 model to 256;
(2) The resulting high-dimensional vectors were reduced via UMAP [50]. UMAP is based on manifold learning and is well suited for handling complex, nonlinear high-dimensional manifold data, while preserving both local neighborhood structure and global structure. The main hyperparameters of the UMAP model are set as follows: n_neighbors = 20, n_components = 5, min_dist = 0.05, metric = ‘cosine’;
(3) The reduced embeddings were clustered using HDBSCAN [51], a density-based clustering method that also provides a hierarchical structure. The key hyperparameters of the HDBSCAN model were configured as follows: min_cluster_size = 50, min_samples = 15, and metric = ‘euclidean’;
(4) Topic labels were extracted using the c-TF-IDF algorithm [28]. First, for the tweet vocabulary obtained through tokenization with the MeCab tool, we applied a standard Japanese stop-word list to remove common stop words. The remaining terms were subsequently ranked by frequency, then three Japanese language experts conducted an iterative manual screening of high-frequency terms in light of corpus characteristics, removing functional words and items without substantive semantic contribution to construct the final vocabulary. This final vocabulary was subsequently used as input for c-TF-IDF to extract topic labels. To reduce noise and prevent high-frequency terms shared across topics from dominating topic representations, we set min_df = 10, max_df = 0.7, and adopted an ngram_range of (1,2);
(5) GPT-4o-mini was employed to generate concise and semantically precise topic summaries. The prompt used for GPT-4o-mini was: “You are an academic assistant. Give a brief topic description based strictly on the provided keywords. Do not introduce new concepts or interpretations”;
(6) These summaries, aligned with the original tweets, were then processed through the XLM-RoBERTa model [52] to map them to the lowest hierarchical layer, resulting in 62 fine-grained topics. This hierarchical mapping links each topic level to the original tweets, enabling a comprehensive transition from raw text to structured hierarchical labels and facilitating detailed analysis of topic evolution.

#### Topic evolution analysis.

Topic evolution analysis aims to reveal the dynamic changes of topics over time, events, or environmental shifts. We adopted the method proposed by Wei et al.[18], which analyses topic evolution based on semantic similarity, to conduct an integrative study of China-related topics on Japanese social media.

Specifically, topic keywords were initially extracted using the c-TF-IDF algorithm. Keywords from 2010 were selected as the “starting words,” and for each subsequent year, the semantic similarity between the current year’s keywords and the previous year’s keywords was calculated. The five keywords most similar to the previous year were selected to represent the evolution of the topic during that period. In this study, topic labels were converted into vectors using the paraphrase-multilingual-MiniLM-L12-v2 model, and cosine similarity was used to measure the semantic relationships between keywords, as expressed by the following formula:


$$Cosine\;Similarity\;(A,B)=\frac{A\cdot B}{∥A∥∥B∥}$$ (1)

Here, A and B represent the vector embeddings of two topic keywords, ∙ denotes the dot product, and ||A|| ||B|| represents the norms of vectors A and B. Cosine similarity is used to measure the similarity between two topics.

By tracing the temporal evolution of topics and quantifying their discussion intensity, we quantified topic fluctuations and integrated dispersed information into a coherent narrative, thereby elucidating the evolution and underlying drivers of Japanese public discourse on China.

#### Sentiment evolution analysis.

To investigate the distribution and evolution of sentiment in Japanese discussions on China, we employed the multilingual-sentiment-analysis model [53] to assess the sentiment of each tweet. This model is based on the mBERT architecture and pre-trained on large-scale multilingual corpora, enabling it to capture fine-grained sentiment features with high accuracy. The model classifies each tweet into five categories: very negative, negative, neutral, positive, or very positive, and provides a confidence score. Using these classifications, we analysed the sentiment distribution for each topic, thereby capturing the evolution and fluctuation of public emotions within each topic.

To visualize the temporal dynamics of Japanese public discourse on China, we employed sentiment heatmaps alongside stacked charts of topic-sentiment evolution. By integrating time, topic distribution, and sentiment polarity, our analysis provides a dynamic perspective on sentiment, revealing fluctuations within the same topic over different periods and comparisons of sentiment across topics at the same point in time. Through sentiment evolution analysis, we aim to capture subtle trajectories in Japanese attitudes toward China, providing robust empirical evidence for understanding the dynamic evolution of public opinion underpinning Sino-Japanese relations.

## Results

The study results include: (1) an overview of hierarchical topic structure; (2) a topic evolution view integrating fragmented information; and (3) sentiment distribution charts and topic-sentiment linkage analysis.

### Overview of hierarchical topic structure

**Fig 2:**
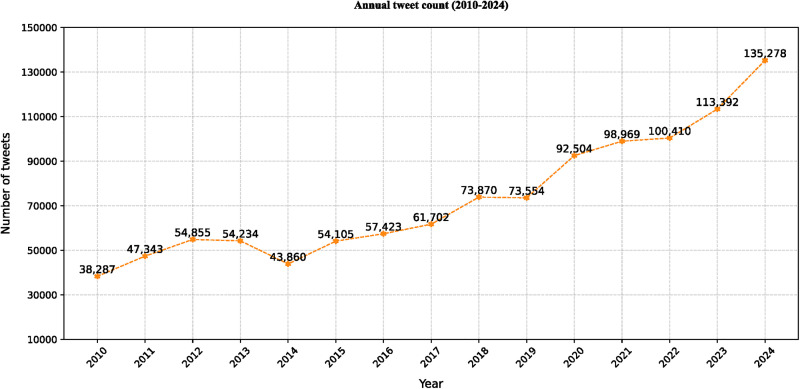
Annual number of China-related tweets.

**Fig 3:**
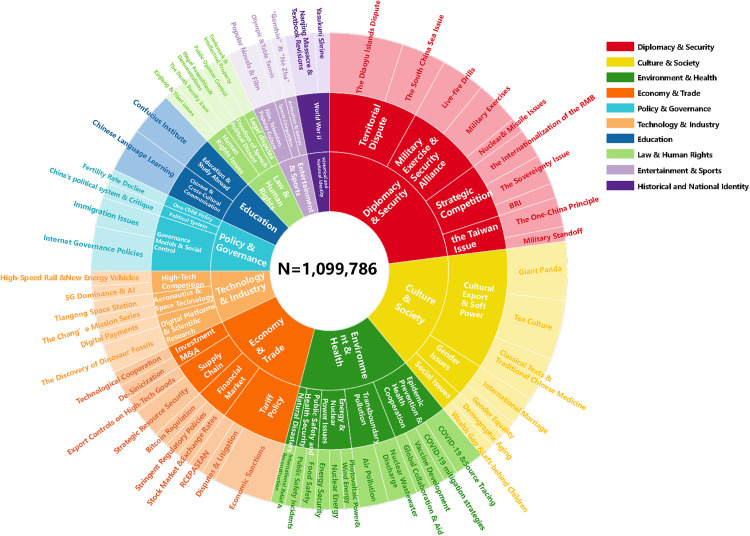
A hierarchical thematic structure of China-related discourse on Japanese twitter.

To address our first research question (RQ1), the distribution and temporal evolution of China-related topics on Japanese social media, we analysed all relevant Japanese tweets from 2010 to 2024, counted the annual number of tweets (Fig 2), and clustered them into a hierarchical topic structure (Fig 3). As shown in Fig 2, the total number of China-related tweets increased steadily over time. Fig 3 presents a sunflower chart of the hierarchical topic architecture, displaying a three-layered structure. The top layer consists of 10 core topics: “Diplomacy & Security,” “Culture & Society,” “Environment & Health,” “Economy & Trade,” “Policy & Governance,” “Technology & Industry,” “Education,” “Law & Human Rights,” “Entertainment & Sports,” and “Historical and National Identity.” The bottom layer clearly presents 62 fine-grained topics, covering mainstream public discourse as well as smaller but critical topics such as AI achievements, traditional Chinese medicine, and nuclear energy. This figure illustrates the hierarchical structure of China-related discourse on Japanese social media, visually conveying the categories and nested relationships among topics.

Table 1 presents the codebook for the ten core China-related topics on Japanese social media. It describes the topics within each top-level category and provides example tweets for each category. A detailed analysis of the topics within the hierarchical structure is presented in the Discussion section.

**Table 1:** The codebook of China-related discourse on the ten topics.

Topics	Percentage	Definition	Example Tweets
**Diplomacy &** **Security**	**23.68%**	**This topic refers to diplomatic interactions, military deployments, and geopolitical strategies between China and Japan, as well as within the broader regional security landscape.**	**“中国漁船団に捕獲してもらいましょう。RT @suka1975: 捕獲しなければどっかに流れて行くでしょう。”**
**Culture &** **Society**	**15.24%**	**This topic refers to perceptions of China’s cultural image, social structure, and cross-cultural interactions.**	**“昨夜NHKで 中国に渡った上野生まれのパンダ シャンシャンを見て 今更ファンになる､”**
**Environment &Health**	**15.60%**	**This topic refers to China’s environmental governance, pandemic control, and energy policies.**	**“中国の新型コロナウイルスの肺炎。来月あたり中国旧正月だよね？日本にもたくさん中国人くるよね？新型ウイルスって治療薬あんの？”**
**Economy &** **Trade**	**15.35%**	**This topic refers to discussions of Sino-Japanese bilateral economic and trade relations, regional economic interdependence, and global market dynamics.**	**“国際通貨基金（ＩＭＦ）の見通しによると、中国の名目国内総生産（ＧＤＰ）が今年には日本を抜いて世界第２位になる、とのこと。”**
**Policy &** **Governance**	**5.97%**	**This topic refers to China’s governance model, policy implementation, and institutional structures, covering a wide range of issues from family planning to political systems and media control.**	**“一人っ子政策のおかげで人口激減が必至ですからね。”**
**Technology& Industry**	**7.08%**	**This topic centers on Japanese users’ collective perceptions and discourses regarding China’s technological development and industrial transformation.**	**“日本の生成AI技術が衰退したら任天堂と提携してる中国のテンセントやアリババの生成AI技術が参入してくるだけだと、反AIさん本当に理解しないな。”**
**Education**	**5.40%**	**This topic refers to China’s education system, language promotion, and study-abroad exchanges, Confucius Institutes and the surge in Chinese language learning.**	**“孔子学院は立命館が第一号で、桜美林・山梨学院など全国に15校程ある模様。”**
**Law &** **Human Rights**	**4.43%**	**This topic focuses on China’s legal system, human rights conditions, reflecting Japanese users’ critical concerns regarding China’s human rights governance and judicial independence.**	**“香港デモの途中まで、俺が多少香港警察に同情的というか、キッズに舐めた真似されてるのによくガマンしてるなあ、デモ隊側の被害者アピールもひどいなあと思った一因に「日本の警察のほうが本気出したら暴力的」というのはあった。”**
**Entertainment& Sports**	**4.24%**	**This topic focuses on China’s film and television, idols and popular culture exchanges, as well as sports competitions.**	**“原神を最初に知ったのはブレワイのパクリ記事からで、開発運営が中国だったので、かなり警戒してたけど、メーカーの事を調べたら、かなりオタク寄りの考えを持っていたので、萌え至上主義の私としては良し良しである** **(-᷅ ·̫ -᷄)** **。”**
**Historical and** **National Identity**	**3.01%**	**This topic refers to historical events and memories related to China and Japan, encompassing issues such as war responsibility and cultural heritage.**	**“南京大虐殺を巡る日中間の共同研究。結局、３０万人以上の中国側と、２０万人までの日本側と、学問上の論争に決着つかず。まあ、無かったと言ってる人もいますけどね。”**

**Fig 4:**
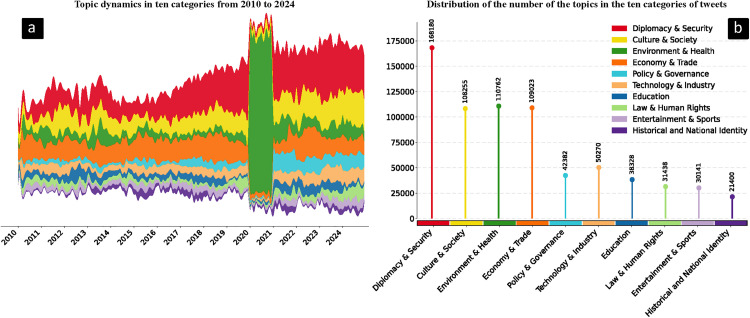
Temporal trends and topic distribution of China-related discourse on Japanese twitter. **a** Temporal trends showing the volume of tweets across the ten core topics over time; **b** Distribution of the ten topics ranked by tweet volume from highest to lowest.

In addition to the fine-grained hierarchical analysis, the temporal trends in topic discussion intensity provide rich insights. As shown in the stream and bar charts (Fig 4), the annual number of tweets related to the ten core China-related topics on Japanese social media is unevenly distributed over time, exhibiting distinct peaks. Fig 4a shows that the discussion intensity of the “Diplomacy & Security” topic increased annually, peaking in 2019; the “Environment & Health” topic experienced a significant surge in 2020 due to the pandemic. “Culture & Society”, “Economy & Trade” and “Technology & Industry” showed a fluctuating upward trend since 2021, reflecting the gradual increase in the visibility of these topics in Japanese public opinion alongside China’s rapid economic development.

Fig 4b illustrates a pronounced long-tail distribution across topic categories [54]. The four topics “Diplomacy & Security”, “Environment & Health”, “Economy & Trade”, and “Culture & Society” dominate discussions, together comprising 69.87% of all tweets. The remaining six topics show comparatively balanced tweet volumes, with “Historical and National Identity” being the least represented. This long-tail distribution of China-related topics on Japanese social media indicates that although public attention is primarily focused on the four major topics, niche topics such as “Entertainment & Sports” and “Historical and National Identity” should not be overlooked.

### Topic evolution view

**Fig 5:**
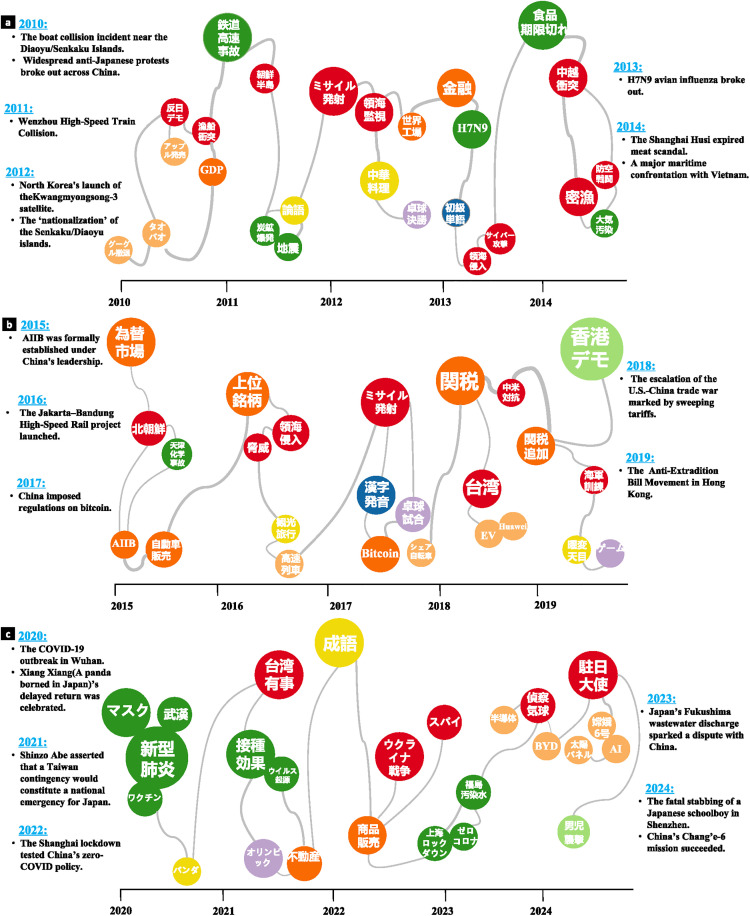
Topic evolution view of China-related discourse on Japanese twitter (2010-2024). **a** shows period from 2010-2014, using the topic “Google’s exit from the Chinese market” as an example. **b** shows period from 2015-2019, While **c** shows period from 2020-2024. The same color represents topics belonging to the same category within the ten topics of China-related discourse. The x-axis in the figure represents the timeline, with the proximity of topic bubbles indicating the degree of semantic relatedness between topics. The size of the bubble represents the number of topics. The bigger the bubble representing a topic is distributed in the graph, the larger its relative quantity. Note that this graph is a schematic, and the time and quantity do not represent precise values.

This study integrates fragmented public discourse data into a coherent and easily interpretable narrative view. Fig 5a–5c illustrate the evolution of China-related topics on Japanese social media from 2010 to 2024, contextualized with major China-related events for each year. The horizontal axis represents time. Bubbles of different colors denote different topics, the bubble size represents the discussion intensity of the corresponding topic, with the radius determined by the normalized number of tweets contained within that topic. The distance between bubbles reflects the degree of correlation between topics. We measured topic correlations using the Spearman correlation coefficient [55]. Spearman’s ρ was computed at a significance level of α = 0.05, and converted to distances using the mapping function $d_{ij}=\text{1}-\rho_{ij}$. These distances were then normalized and used to position the bubbles. The smaller the distance between two bubbles, the stronger the correlation between the corresponding topics.

We applied HDBSCAN (with parameters min_cluster_size = 15, min_samples = 5, and metric = ‘euclidean’) to the tweet embeddings within each topic for a second-stage clustering to identify event-level subclusters that are both semantically cohesive and temporally concentrated, thereby extracting specific events. Event intensity was then calculated based on the number of tweets and the temporal window as:


$$EventIntensity=\frac{N}{\Delta t}$$ (2)

where  N denotes the number of tweets in a given event and Δt represents its duration. Based on this metric, one or two high-intensity representative events were selected for each year as major events.

Using the 2010 keyword “Google exits China” (「グーグル撤退」) as the initial point of the evolution pathway, we employed semantic similarity clustering to identify the five most semantically related keywords, such as “Taobao” (「タオバオ」) and “Apple launch” (「アップル発売」). Keywords from the latest timestamp in the previous year subsequently evolve into the five keywords for the following year, continuing iteratively.

As shown in Fig 5, “Diplomacy & Security” (red) consistently appears and maintains a prominent position from 2010 to 2024. Key issues within this topic include the Taiwan issue, North Korea’s nuclear program, China–Vietnam disputes in the South China Sea, the Sino–Japanese Diaoyu/Senkaku Islands dispute, and Sino–U.S. confrontation. These discussions are typically triggered by major diplomatic events and historical issues, such as the 2010 detention of a Chinese fishing boat by Japan, which sparked large-scale anti-Japan protests in China; North Korea’s missile launch and Japan’s “nationalization” of the Diaoyu/Senkaku Islands in 2012; China’s intensified East China Sea patrols in 2013; and the escalation of Sino–U.S. strategic competition in 2018.

The “Economy & Trade” (orange-red) and “Technology & Industry” (creamy orange) topics exhibit strong associations with “Diplomacy & Security”. Early discussions focused on economic scale and market opportunities, including events such as China surpassing Japan in nominal GDP in 2010, Taobao reaching 370 million members, and the launch of the iPhone 4 in China. Subsequently, technological innovation and outbound investment became focal points, including the establishment of the Asian Infrastructure Investment Bank (2015), China’s high-speed rail expansion abroad (2016), cryptocurrency regulation in China (2017), Huawei and BYD’s overseas developments (2018, 2023), and breakthroughs in AI technology (2024).

The “Environment & Health” (green) topic encompasses narratives related to accidents, epidemics, and food safety, with discussion peaks largely triggered by public safety incidents, including the Wenzhou train collision (2011), the H7N9 avian influenza outbreak (2013), the Fuxi food contamination scandal (2014), and the COVID-19 pandemic (2020). By contrast, topics such as “Culture & Society”, “Law & Human Rights”, “Education”, and “Entertainment & Sports” maintained relatively stable discussion levels, centering on cultural exchange, Chinese language learning, sporting events, and select societal incidents.

### Sentiment distribution

To explore the sentiment distribution and evolution patterns in China-related discourse on Japanese social media (RQ2), this study employed the multilingual- sentiment-analysis pre-trained model to perform fine-grained sentiment classification on the collected China-related tweets, categorizing them into five classes: very negative, negative, neutral, positive, and very positive.

This study found that the overall sentiment distribution of China-related discourse on Japanese social media is presented in Table 2:

**Table 2:** Overall sentiment distribution of China-related discourse on Japanese twitter.

Very Negative	Negative	Neutral	Positive	Very Positive
35.5%	7.0%	27.2%	8.3%	22.0%

Table 2 indicates that the sentiment of Japan’s social media discourse on China is predominantly negative, with very negative and negative sentiments together representing 42.5%, compared to just 30.3% for very positive and positive sentiments, highlighting a clear predominance of unfavourable evaluations over favourable ones.

**Fig 6:**
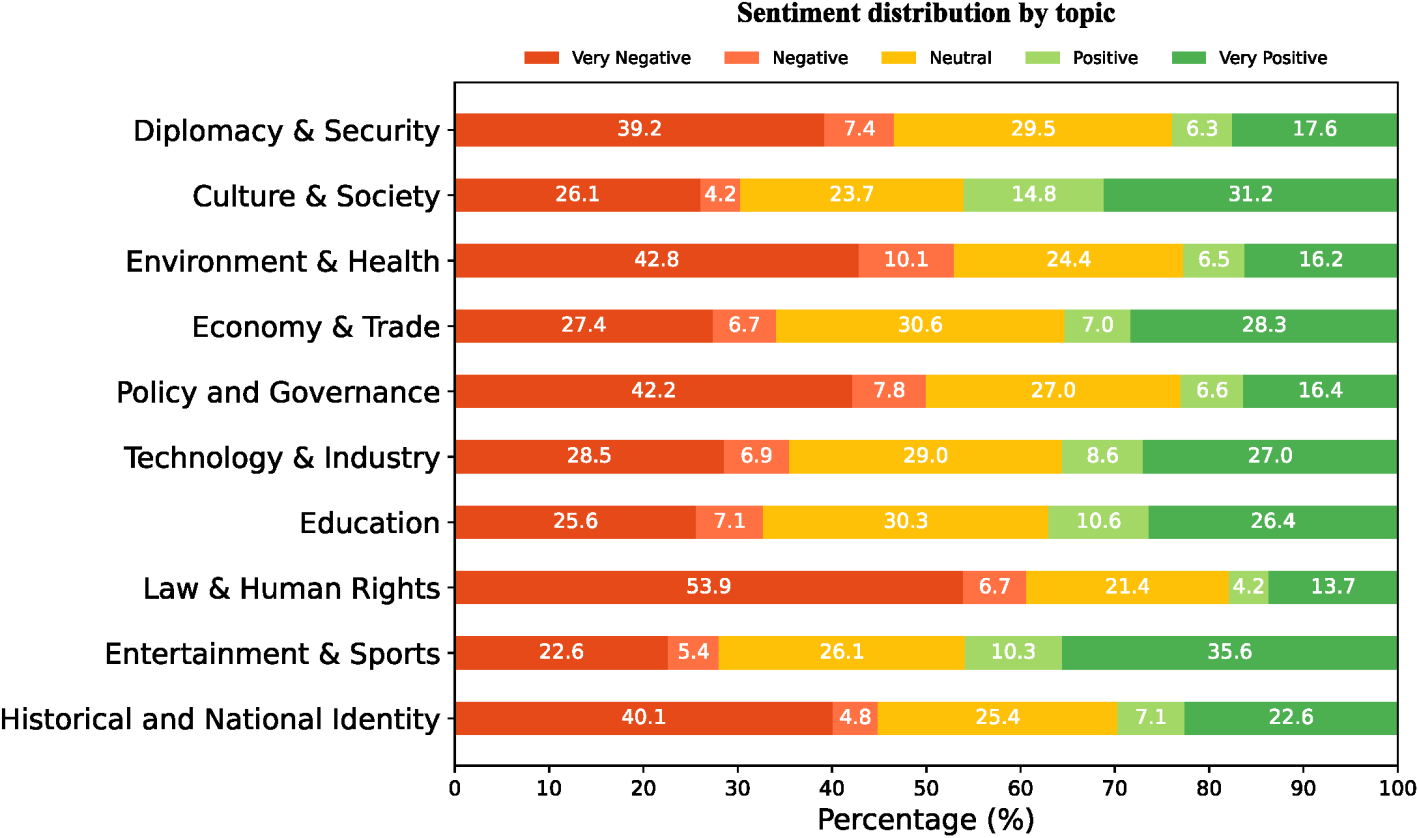
Sentiment distribution across the ten topics. The figures indicate the proportion of tweets in each of the five sentiment categories (very negative, negative, neutral, positive, very positive) as a percentage of the total tweets under each topic.

Fig 6 illustrates the sentiment distribution across the ten core topics. As shown, the proportion of neutral sentiment in each topic is roughly similar, ranging from 20% to 30%, indicating that approximately one quarter of China-related discussions maintain a relatively neutral tone. In addition, the share of positive and negative sentiments is relatively small across all topics, whereas the proportions of very positive and very negative sentiments are comparatively larger.

As shown in Fig 6, the proportion of very negative sentiment is notably high in five domains: “Diplomacy & Security” (39.2%), “Policy & Governance” (42.2%), “Environment & Health” (42.8%), “Law & Human Rights” (53.9%), and “Historical and National Identity” (40.1%). In contrast, the proportion of very positive sentiment is markedly higher in five topics: “Culture & Society” (31.2%), “Economy & Trade” (28.3%), “Technology & Industry” (27.0%), “Education” (26.4%), and “Entertainment & Sports” (35.6%) compared to other domains.

### Topic-sentiment linkage analysis

**Fig 7:**
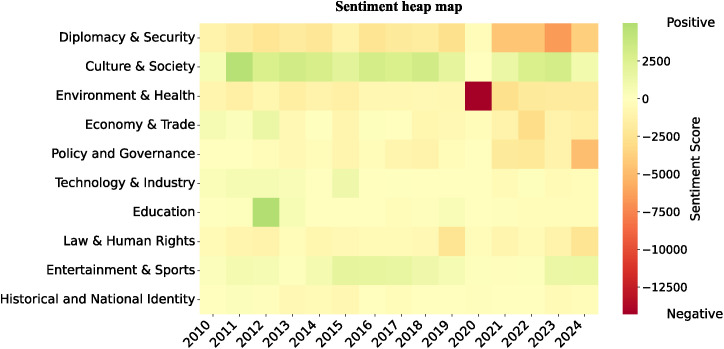
Overall sentiment evolution in China-related discourse on Japanese twitter (2010−2024). It presents a sentiment heatmap organized by year and topic. Color intensity reflects the sentiment score, calculated annually by summing the sentiment values of all tweets within each topic (very negative = −2 negative = −1, neutral = 0, positive = +1, very positive = +2). Darker colors indicate stronger sentiment polarity, whether positive or negative. The horizontal axis represents the year, while the vertical axis lists the ten topics.

**Fig 8:**
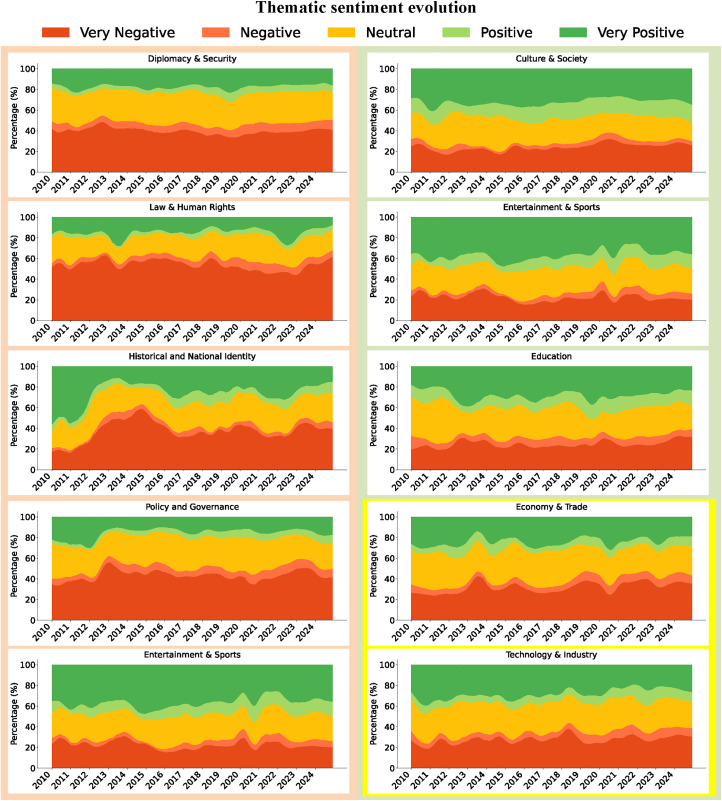
Stacked charts of topic-sentiment evolution (2010-2024). This figure consists of ten subplots, each representing a distinct topic. The ten subplots are divided into two groups: the five on the left represent topics in the domain of high politics, while the five on the right correspond to topics in the domain of low politics. Among the latter, the first three pertain to culture and education, and the final two relate to economics and technology. In each subplot, the horizontal axis denotes time, while the vertical axis displays the stacked area segmented into five colors. Each color corresponds to the proportion of tweets expressing one of five sentiment categories—very negative, negative, neutral, positive, and very positive—relative to the total number of tweets within that topic.

Drawing on the sentiment heatmap (Fig 7) and the stacked charts of topic-sentiment evolution (Fig 8), this study uncovers the temporal dynamics of sentiment across different topics within Japan’s social media discourse on China.

Fig 7 illustrates that sentiment trends diverge markedly across different topics. Specifically, “Diplomacy & Security”, “Law & Human Rights”, “Policy & Governance”, “Environment & Health”, and “Historical and National Identity” exhibit a clear negative shift overall. In contrast, “Culture & Society”, “Education”, and “Entertainment & Sports” maintain a predominantly positive shift, while “Economy & Trade” and “Technology & Industry” fluctuate between positive and negative sentiments.

Fig 8 traces the thematic trajectories in finer detail, corroborating the broader trends outlined above. In the aftermath of Japan’s 2012 “nationalization” of the Diaoyu/Senkaku Islands, negative and very negative sentiments in “Diplomacy & Security” spiked to 54.92%. Similarly, the proportion of negative sentiments within “Historical and National Identity” remained around 60% between 2014 and 2015, peaking at 63.51% in October 2014, highlighting the immediate mobilizing effect of territorial disputes on national identity and collective belonging [56]. In addition, “Environment & Health” experienced a steep surge in negative sentiment during the COVID-19 outbreak in 2020, while “Law & Human Rights” reached a negative sentiment peak during the 2019 Hong Kong anti-extradition bill movement.

At the same time, positive sentiment clustered most prominently within socially interactive topics. In “Entertainment & Sports”, positive and very positive sentiments consistently accounted for 20–40%, while “Culture & Society” maintained a higher range of 30–50%, reflecting a sustained polarization toward positive affect. Notably, during the COVID-19 pandemic in 2020, the share of positive sentiment in “Entertainment & Sports” stayed around 40%, and in October of the same year, following the success of the Chinese mobile game Genshin Impact at the top of Japan’s App Store rankings, positive sentiment rose to 55.41%.

Sentiments within the “Economy & Trade” and “Technology & Industry” fluctuated noticeably in response to policies and events. In “Economy & Trade”, negative sentiment climbed to 47.35% in 2013 amid regional economic tensions, and surged again to 47.86% during the 2018 US–China trade war. Following the signing of the Regional Comprehensive Economic Partnership (RCEP) in 2020, positive sentiment (positive and very positive) rebounded to 39.37%, underscoring the regulatory influence of policy signals on public attitudes. The “Technology & Industry”, though generally rated positively, displayed even sharper fluctuations: In 2018, the US imposed sanctions on Huawei, and Japan promptly removed the company from government procurement lists, driving negative sentiment within this topic to 43.82%. By 2019, however, sentiment had swiftly shifted back to positive.

## Discussion

This study seeks to illuminate the multidimensional complexity and dynamic evolution of Japanese public perceptions and attitudes toward China. Unlike prior research largely dependent on conventional opinion polls, it offers a fine-grained “topic–sentiment” mapping framework. Our data analysis reveals that:

First, the topic modeling results indicate that public attention toward China has generally increased, with a core focus on “Diplomacy & Security”, followed by “Environment & Health”, “Economy & Trade”, and “Culture & Society”. This largely supports Gong & Nagayoshi’s observation that security concerns form the cornerstone of Japanese perceptions of China [7], while also uncovering a more nuanced picture: within “Diplomacy & Security”, the public’s focus extends beyond the Taiwan Strait crisis, military drills, and U.S.–China confrontations to incorporate the “Technology & Industry” topic through discourses of “technological hegemony” and “dual-use technologies.” Moreover, after the pandemic, public attention toward cultural, economic, and technological topics increased significantly, highlighting diversification in public perceptions of China.

Second, sentiment analysis reveals that Japanese attitudes toward China are overall skewed negative, yet neutral sentiment constitutes about one-quarter of responses. Positive and negative sentiments are roughly balanced, with strongly positive and strongly negative sentiments relatively pronounced. Compared to earlier studies [8], the higher prevalence of neutral sentiment here indicates that a significant segment of the public maintains a nuanced, non-polarized stance toward China, rather than being confined to sharply opposing views.

The topic–sentiment linkage analysis reveals that Japanese attitudes exhibit diverse patterns across topics. Highly politicized topics like “Diplomacy & Security” and “Historical and National Identity” are saturated with negative sentiment, acting as flashpoints for digital nationalism. In contrast, cultural and social domains remain largely depoliticized spaces for positive engagement. Economic and technological topics exhibit dynamic ambivalence, framed alternatively as threats or opportunities, while health crises trigger negative sentiments linked to perceptions of governance failure and “risk spillover”. This pattern strongly indicates that opinion formation is a dual process, driven by external events but mediated through topic-specific affective affordances.

Overall, this study moves beyond the binary framing of “positive–negative” attitudes by conceptualizing Japanese perceptions of China as a communicative process unfolding within the digital public sphere [57,58]. The findings show that public opinion is not simply a reflection of geopolitical events but a discursive formation in which topics such as diplomacy, culture, health, and technology serve as sites of affective negotiation [59]. Social media thus constitutes a hybrid arena where digital nationalism and everyday discourse intersect, shaping, contesting, and reconfiguring China’s image over time. This perspective highlights digital media not merely as a channel of information exchange but as a constitutive space for public diplomacy and transnational identity-making in East Asia.

By combining fine-grained sentiment analysis with BERTopic, this study expands the theoretical boundaries of research on Japan’s China-related public opinion by introducing a dynamic socio-cognitive perspective. We demonstrate that Japanese public attitudes toward China are not merely static reflections of individual experience but evolve through the interplay of major events, user engagement, and discourse framing. This analytical framework elucidates the temporal dynamics of topic prominence and the associated emotional valences, thereby offering methodological and theoretical guidance for examining China-related discourse on social media. On one hand, the framework enables researchers to extract events from large-scale social media data in a hierarchical manner and integrate the fragmented information into a coherent narrative. On the other hand, it allows researchers to identify critical points of emotional shift, examine connections between different topics, and anticipate emerging public opinion risks, thereby informing policy assessment and optimization.

Based on the above findings, this study offers multiple insights for the management of public opinion and public diplomacy in Sino–Japanese relations. First, given the thematic variation in public sentiment, policymakers should precisely identify and address issue-specific concerns, implementing tailored communication and engagement strategies. Second, security concerns constitute a central consideration in China’s proactive outreach to Japan. Integrating narratives of “cooperative security” and “mutual trust” into non-security domains (such as environmental governance or renewable energy cooperation) can function as a buffer to mediate between conflict and collaboration. Third, the complexity of Japanese perceptions toward China calls for coordinated, multi-platform and multi-actor initiatives to amplify positive narratives. For instance, Chinese residents in Japan, international students, online influencers, and Japanese creators could collaboratively produce short videos highlighting social interactions, thereby reinforcing the dissemination of positive sentiment. Fourth, in sudden public health or environmental crises, China should strengthen risk communication within public diplomacy, ensuring information is released in a timely, transparent, and credible manner to mitigate the sudden surge of negative sentiment.

## Limitations

Although our study addresses the two research questions, several limitations remain. First, public opinions expressed on social media may be subject to sampling bias. According to a 2024 survey by Japan’s Ministry of Internal Affairs and Communications, Twitter users aged 20–29 constitute the largest share at 81.6%, followed by adolescents aged 10–19 (65.7%) and adults aged 30–39 (61.0%), whereas participation among the 40–60 age group remains comparatively low [60]. Consequently, the social media data used in this study may primarily reflect the perspectives of younger users, urban residents, or individuals with particular interests, whereas older adults, low-income individuals, and users in remote regions are underrepresented, potentially introducing sampling bias. This sampling bias may limit generalizability. Specifically, it may amplify the views and emotional expressions of digitally active users, thereby constraining a comprehensive understanding of China-related attitudes across different segments of Japanese society. Second, the collection of tweet data relied on pre-defined keyword searches. Although this approach is highly targeted and operationally feasible, it may omit content closely related to the research themes that does not explicitly contain the selected keywords. Future studies could combine multi-level keywords and semantic expansion dictionaries to further enhance data coverage. Third, although the sentiment analysis model and LLM-assisted topic labeling methods used in this study are well-established in current text-mining research, their pre-trained nature inevitably entails a “black-box” characteristic. This includes potential biases in the training corpus and the non-transparent reasoning mechanisms, which may introduce errors in sentiment recognition and topic labeling and pose fairness and ethical risks [61]. Nevertheless, these errors primarily affect model output precision without altering overall topic trends or study conclusions, which remain robust.

## Conclusion

Drawing on tweets related to China on Japan’s Twitter platform from 2010 to 2024, this study provides a long-term, multi-level, and fine-grained account of Japanese perceptions and attitudes toward China. The research contributes to the field in three ways. First, by combining large-scale social media data with detailed “topic–sentiment” analysis, it transcends the conventional quantitative approach reliant on opinion surveys, uncovering differentiated patterns and evolution trajectories of Japanese attitudes across distinct topics, thereby providing a more explanatory framework for studying Japanese public opinion. Second, with its large sample size and extended temporal coverage, the study offers a systematic, dynamic perspective spanning macro- to micro-level analyses. Third, it is the first to integrate BERTopic topic modeling with large-scale sentiment analysis, examining the coupled evolution of topics and sentiments in Japan-related social media discourse on China, and providing methodological tools and analytical frameworks that can guide future research on public opinion.

Future research could be expanded in several directions. First, Future research may incorporate demographic variables such as gender and age within permissible data constraints to examine differences in expression, emotional patterns, and interaction behaviors across groups, thereby providing a more comprehensive understanding of public attitude formation mechanisms. Second, cross-national comparative studies could examine how China-related discourse is constructed on social media platforms in Japan versus countries such as the UK and the US, shedding light on the cultural, social, and institutional mechanisms underlying these differences. Third, future research could build on the analytical framework of this study by incorporating domain expert knowledge to construct an ontology tailored to Japan-related Chinese discourse on social media, and integrate it with LLM-driven text classification and topic identification pipelines to enhance the robustness and interpretability of the analysis across contexts and events. Technically, the hyper-knowledge graph system introduced by Chen et al. offers a useful model for structuring complex text analysis outputs into a queryable and inferable knowledge system [62]. Following this approach, future work could map tweets, topics, sentiment orientations, and their temporal evolution into nodes and edges within a graph database, offering a potential framework for supporting policy analysis and public opinion research.
